# Different non-synonymous polymorphisms modulate the interaction of the WRN protein to its protein partners and its enzymatic activities

**DOI:** 10.18632/oncotarget.13341

**Published:** 2016-11-14

**Authors:** Jean-Philippe Gagné, Sophie Lachapelle, Chantal Garand, Serges P. Tsofack, Yan Coulombe, Marie-Christine Caron, Guy G. Poirier, Jean-Yves Masson, Michel Lebel

**Affiliations:** ^1^ Centre de Recherche du CHU de Québec, Pavillon CHUL Université Laval, Faculté de Médecine, Québec, Canada; ^2^ Centre de Recherche sur le Cancer de l’Université Laval, Hôpital Hôtel-Dieu de Québec, Québec, Québec, Canada

**Keywords:** mass spectrometry, proteomics, werner syndrome, polymorphism, exonuclease, Gerotarget

## Abstract

Werner syndrome (WS) is characterized by the premature onset of several age-associated pathologies including cancer. The protein defective in WS patients (WRN) is a helicase/exonuclease involved in DNA replication and repair. Here, we present the results of a large-scale proteome analysis that has been undertaken to determine protein partners of different polymorphic WRN proteins found with relatively high prevalence in the human population. We expressed different fluorescently tagged-WRN (eYFP-WRN) variants in human 293 embryonic kidney cells (HEK293) and used a combination of affinity-purification and mass spectrometry to identify different compositions of WRN-associated protein complexes. We found that a WRN variant containing a phenylalanine residue at position 1074 and an arginine at position 1367 (eYFP-WRN(F-R)) possesses more affinity for DNA-PKc, KU86, KU70, and PARP1 than a variant containing a leucine at position 1074 and a cysteine at position 1367 (eYFP-WRN(L-C)). Such results were confirmed in a WRN-deficient background using WS fibroblasts. Interestingly, the exonuclase activity of WRN recovered from immunoprecipitated eYFP-WRN(L-C) variant was lower than the eYFP-WRN(F-R) in WS cells. Finally, HEK293 cells and WS fibroblasts overexpressing the eYFP-WRN(F-R) variant were more resistant to the benzene metabolite hydroquinone than cells expressing the eYFP-WRN(L-C) variant. These results indicate that the protein-protein interaction landscape of WRN is subject to modulation by polymorphic amino acids, a characteristic associated with distinctive cell survival outcome.

## INTRODUCTION

Werner Syndrome (WS; MIM number #277700) is a rare autosomal recessive disorder that displays many of the clinical symptoms of aging at an early age. From their second decade of life onward, WS patients develop pathologies that prematurely resemble many traits of normal aging such as osteoporosis, ocular cataracts, graying and loss of hair, diabetes mellitus, arteriosclerosis, and cancer [1–4]. Death generally occurs in the fifth decade of life from heart demise or cancer. Accumulating evidences indicate that the WRN (MIM number 604611) encoded gene product is a suppressor of illegitimate DNA recombination. Indeed, WS cells are characterized by the presence of deletions and variegated chromosomal translocations [5, 6]. Processes such as DNA replication or transcription generate regions of single-stranded DNA, which may inadvertently provide a substrate for the initiation of recombination. Various mechanisms have evolved to ensure that recombination does not occur promiscuously during these processes and the WRN protein may be part of one such mechanism. Thus, one potential role for WRN would be to actually monitor recombinational repair of double-strand breaks [7]. During the process of recombination, nonhomologous regions of DNA could inadvertently be used as templates for repair. WRN protein will not inhibit the initiation of recombination but will dissociate abnormal recombination intermediates [8–10]. Accordingly, purified WRN protein has an affinity for DNA fork structures such as those observed during DNA recombination [11]. Furthermore, WRN can migrate Holliday junctions (a recombination intermediate) [8]. Thus, a mutation in WRN may lead to an increased frequency of illegitimate recombination during the repair of breaks at transcriptional sites or DNA replication forks, creating small deletions or variegated chromosomal translocations. In addition to homologous recombination, WRN is involved with the KU70/86 complex and DNA-PKc in nonhomologous end joining reactions [12, 13]. Finally, the interplay between poly(ADP-ribose) polymerase 1 (PARP1) and WRN plays an essential role in maintaining genome integrity in eukaryotic cells [14, 15], notably by suppressing oxidized DNA in long patch base excision pathway [16, 17]. Thus, WRN is likely to be involved in multiple DNA repair pathways. In the absence of a functional WRN protein, accumulation of deletions and translocations could potentially inactivate tumor suppressor genes or activate oncogenes, accelerating tumor formation and/or aggressiveness. The most frequent cancers in WS patients are thyroid neoplasms, malignant melanoma, meningioma, soft tissue sarcomas, leukemia and pre-leukemic conditions of the bone marrow [18].

**Figure 1:**
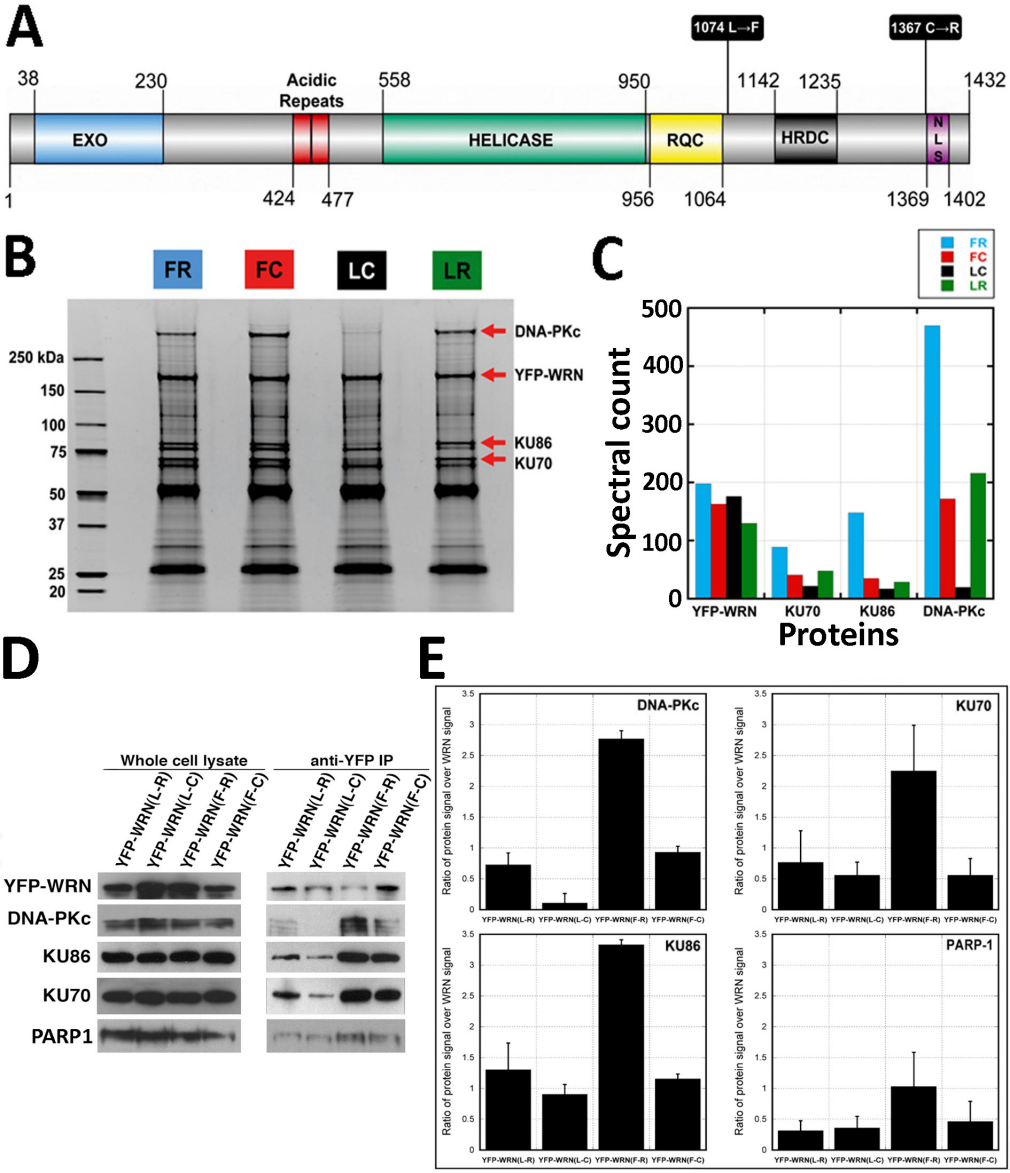
Co-purification of WRN-interacting proteins Affinity-purification of eYFP-tagged WRN variants was performed with antibody-coupled magnetic beads in HEK293 whole cell extracts. **A.** Schematic representation of the human WRN protein with the different polymorphic residues targeted in this study. C = cysteine; F = phenylalanine; L = leucine; R = arginine. Domain boundaries were drawn according to Uniprot (UniProtKB - Q14191) and [56]. **B.** Protein profiles of WRN-interacting proteins. Immunoprecipitation extracts obtained with each eYFP-WRN variants were resolved on SDS-PAGE and stained with SYPRO. All immunoprecipitations were performed 24 h after the transfection of eYFP-WRN expressing vectors. Molecular-mass sizes are indicated in kilodaltons (kDa). Arrows point to bands identified as major eYFP-WRN variants: DNA-PKc, KU86, and KU70 proteins. **C.** Diagram showing a summary of the spectral counts for YFP-WRN, DNA-PKc, KU86, and KU70 proteins from the tandem mass spectrometry analysis of protein extracts shown in B. **D.** Selected WRN-interacting proteins found by mass spectrometry analysis in eYFP-WRN immunoprecipitation extracts (anti-YFP IP) from transfected HEK293 cells were validated by Western blot analysis. A representative Western blot is shown with antibodies against DNA-PKc, KU86, KU70, and PARP1. **E.** Semi-quantitative analysis of protein abundance in eYFP-WRN immunoprecipitation extracts. The enrichment ratios of the indicated co-immunoprecipitated protein signal over the immunoprecipitated eYFP-WRN signal are indicated in the histograms. All co-immunoprecipitation analyses were performed in duplicate. Error bars (standard error of the mean (SEM)) are indicated for each histogram.

Despite the rarity of WS, several common polymorphisms in the WRN gene have been associated with different cancers in specific populations. The two most common polymorphisms (most common in the general population) that have been analyzed are single nucleotide polymorphisms (SNPs) that substitute amino acid 1074 (Leu to Phe) or amino acid 1367 (Cys to Arg) of the WRN coding sequence. If we consider the helicase domain as being in the middle of the WRN protein sequence, each of these changes are located C-terminal to the DNA helicase catalytic domain (Figure 1A). It has been reported that the Arg allele at position 1367 of the WRN gene product is protective against bone and soft tissue sarcomas in Japan [19]. Similarly, the same Arg allele was associated with a lower risk of non-Hodgkin lymphoma among women in the state of Connecticut in the USA [20]. Although some studies suggested the lack of correlation between the Arg1367 allele and the development of non-Hodgkin lymphoma [21, 22], a meta-analysis of several genome-wide association studies has rather indicated a relationship between the Arg1367 allele and the incidence of this lymphoma [23].

Several other studies have indicated an association between the Arg1367 allele of the WRN gene product and different cancer types. For example, the Arg/Arg genotype was associated with higher mean age of onset of gastric cancer in Brazil and a higher risk of esophageal cancer in China [24, 25]. In two studies, one Arg1367 allele was sufficient to increase the risk of developing breast cancer among German and American women [26, 27]. In contrast, Chinese women with a Phe/Phe genotype at position 1074 of the WRN protein showed an increased risk of developing breast cancer but no association was found between the Arg1367 variant and breast cancer in the same population [28]. Finally, the Phe/Phe genotype at position 1074 of the WRN protein is associated with increased risk of lung cancer in the UK [29] and Chinese subjects with one Leu1074 variant is associated with a lower prevalence of prostate cancer [30]. The contradictory results between these reports may be due to the different sample size under study. Furthermore, population stratification and ethnic considerations influence the interpretation of the association results.

Interestingly, haplotypic analyses on different WRN coding SNPs indicated that Chinese workers exposed to benzene were more at risk of developing hematotoxicity depending on their polymorphisms [31, 32]. It was concluded that the combination of different SNPs at both amino acid positions 1074 and 1367 of the WRN protein had broad effects on the count of several white blood cell subtypes upon benzene-induced hematotoxicity. This latter work is an example of the few reports analyzing SNP combinations of the WRN gene in association studies.

The exact cellular impact of these polymorphisms in the WRN gene and how they contribute to chemical toxicity and cancer predisposition remains rudimentary. Although the WRN Cys1367Arg substitution is located close to the nuclear localization signal motif, no difference in nuclear localization could be detected between the Cys1367 and the Arg1367 WRN variants [33]. Furthermore, no significant difference could be detected between the Arg1367 and the Cys1367 purified WRN variants or the Leu1074 and the Phe1074 variants with regards to *in vitro* exonuclease or helicase activities [34]. One possibility is that these variants may affect the interaction of the WRN gene product with other nuclear proteins. In this study, we show for the first time that different polymorphic WRN protein variants have distinct macromolecular protein complexes composition as a consequence of altered physical affinity for various DNA damage response factors. Such changes in the local environment of WRN may directly modulate its activity towards alternate or damaged DNA structures.

## RESULTS

### Identification of WRN-interacting proteins by mass spectrometry

To identify multi-protein complexes specifically associated with different WRN polymorphic variants, we transfected human 293 embryonic kidney cells with eYFP-WRN expression constructs containing the Leu1074-Cys1367, Phe1074-Cys1367, Leu1074-Arg1367, or Phe1074-Arg1367 variant. The numbers represent the position of the indicated amino acids in the different WRN protein variants (Figure 1A). There are many advantages to the use of eYFP-WRN chimera in our proteome-wide analysis. The YFP-tag confers a strong affinity to commercially available antibodies against eYFP for large-scale immunoprecipitation experiments. N-terminal eYFP tagging of WRN have minimal effect on its sub-cellular localization, tertiary structure or biological activity, and thus recapitulates the dynamics of the endogenous WRN protein. In addition, the overexpression of eYFP-WRN to facilitate its isolation in whole-cell extracts is a very effective strategy that has previously been performed to identify WRN-interacting proteins [35].

For the affinity-purification of WRN-associated protein complexes, immunoprecipitation assays were carried out under rather mild detergent and ionic strength that allowed efficient isolation of intact protein complexes (see Materials and Methods). Broad specificity DNAse and RNAses were added in the extraction buffer to favor the identification of direct protein-protein interactions and to minimize protein/nucleic acid/WRN interactions. The immunoprecipitated proteins were resolved by SDS-PAGE and stained with Sypro Ruby (Figure 1B). The entire protein eluate resolved by SDS-PAGE was extracted for a complete coverage of the co-immunoprecipitated proteins rather than limited to high-abundance protein bands. Protein tracks were cut into several slices for in-gel trypsin digestion followed by liquid chromatography tandem mass spectrometry (LC-MS/MS). Using stringent protein identification criteria, we could reliably annotate a total of 7677 MS/MS spectra corresponding to 442 unique proteins (data available upon request). Common unspecific proteins (keratins, serum albumin, trypsin, IgGs) and unspecific binding proteins found in control eYFP immunoprecipitation extracts were cut out of the dataset to generate a preliminary listing of 375 potential WRN-interacting proteins (Supplementary Table S1). Since WRN is exclusively a nuclear protein, we finally restricted the remaining of our study to a list containing exclusively nuclear proteins or proteins known to shuttle to the nucleus. Based on gene ontology terms “nucleus” and “DNA metabolic process” (using DAVID), the supplementary Table S1 also indicates 183 nuclear proteins co-immunoprecipitating with the different eYFP-WRN construct variants. Table 1 gives a list of nuclear proteins co-immunoprecipitated with the different eYFP-WRN construct variants with a minimum of two unique peptides assignments. Proteins such as DNA-PKc, TMPO, KU86, KU70, RPA1, PARP1, and RPA2 were among the proteins identified with the highest peptide spectral counts, a parameter that can be used as a semi-quantitative readout of relative protein abundance.

**Table 1:** List of nuclear proteins identified by mass spectrometry interacting with all the different WRN variants

Gene name	Number of unique peptides				Description
	FC*	FR	LC	LR	
PRKDC	172	470	20	216	DNA-dependent protein kinase catalytic subunit
WRN	163	198	176	130	Werner syndrome gene product
TMPO	60	172	51	13	Thymopentin (lamina-associated polypeptide 2)
XRCC5	35	148	17	29	KU86 ATP-dependent DNA helicase 2 subunit 2
XRCC6	41	89	22	48	KU70 ATP-dependent DNA helicase 2 subunit 1
RPA1	22	40	43	21	Replication protein A 70 kDa DNA-binding subunit
PARP1	10	23	9	8	Poly(ADP-ribose) polymerase 1
RPA2	6	19	11	9	Replication protein A 32 kDa subunit
CUX1	8	5	9	9	Homeobox protein cut-like 1
LIG3	5	10	3	2	DNA ligase 3
HNRNPA2B1	6	3	10	2	Heterogeneous nuclear ribonucleoprotein A2/B1
HERC2	3	8	5	7	Hect domain-containing protein 2-E3 ubiquitin ligase
CHD4	6	4	7	6	Chromodomain-helicase-DNA-binding protein 4
HSPD1	3	7	7	2	60 kDa heat shock protein
HIST1H4A	2	5	6	2	Histone H4
VRK3	5	5	6	4	Vaccinia related kinase 3
SATB2	2	4	2	3	DNA-binding protein SATB2

* FC corresponds to YFP-WRN with F amino acid at position 1074 and C at position 1367, FR corresponds to YFP-WRN with F at position 1074 and R at position 1367, LC corresponds to YFP-WRN with L at position 1074 and C at position 1367, LR corresponds to YFP-WRN with L at position 1074 and R at position 1367. F=phenylalanine, L=leucine, C=cysteine, R=arginine.

One striking observation regarding WRN-interacting proteins is the massive variations of spectral counts relative to the KU70/KU86/DNA-PKc complex involved in the nonhomologous end joining DNA repair pathway (Table 1). The index of spectra assigned to DNA-PKc, KU86, and KU70 correlates with the intensities of the bands shown in the Sypro Ruby staining (arrows in Figure 1B and relative abundance in Figure 1C). To confirm the differential interactions between DNA-PKc, KU86, or KU70 and the different eYFP-WRN variants, HEK 293 cells were first transfected with the different eYFP-WRN expression vectors followed by immunoprecipitation of the eYFP-WRN variants 24 hours later with the anti-eYFP antibody. The immunoprecipitates were analyzed by Western blotting with the appropriate antibodies. As shown in Figure 1D, DNA-PKc was hardly detectable in the immunoprecipitate of the eYFP-WRN(L-C) variant compared to the other three variants (Figure 1D and 1E). It was detected only after a long exposure (45 min) of the immunoblot (supplementary Figure S1A). In contrast, the eYFP-WRN(F-R) is much more proficient to pull-down DNA-PKc and its regulatory KU subunits. More KU86 protein was recovered from the eYFP-WRN(F-R) immunoprecipitation experiments than the other variants (Figure 1D and 1E). The eYFP-WRN(F-R) variant also co-immunoprecipitated more KU70 than the other variants (Figure 1D and 1E).

Since PARP1 was also one of the major DNA repair protein bound to WRN (Table 1), we also examined the amount of PARP1 co-immunoprecipitated with the different variants. As indicated in Figure 1D and 1E, the eYFP-WRN(F-R) variant co-immunoprecipitated more PARP1 than the other variants. These results are consistent with spectra count-based estimates of the LC-MS/MS analysis (Table 1). Together, these results indicate that different polymorphic amino acids in WRN affect its affinity to DNA-PKc, PARP1 and the KU complex, which are major components of DNA damage response.

### Enzymatic activities of two eYFP-WRN polymorphic variants

**Figure 2:**
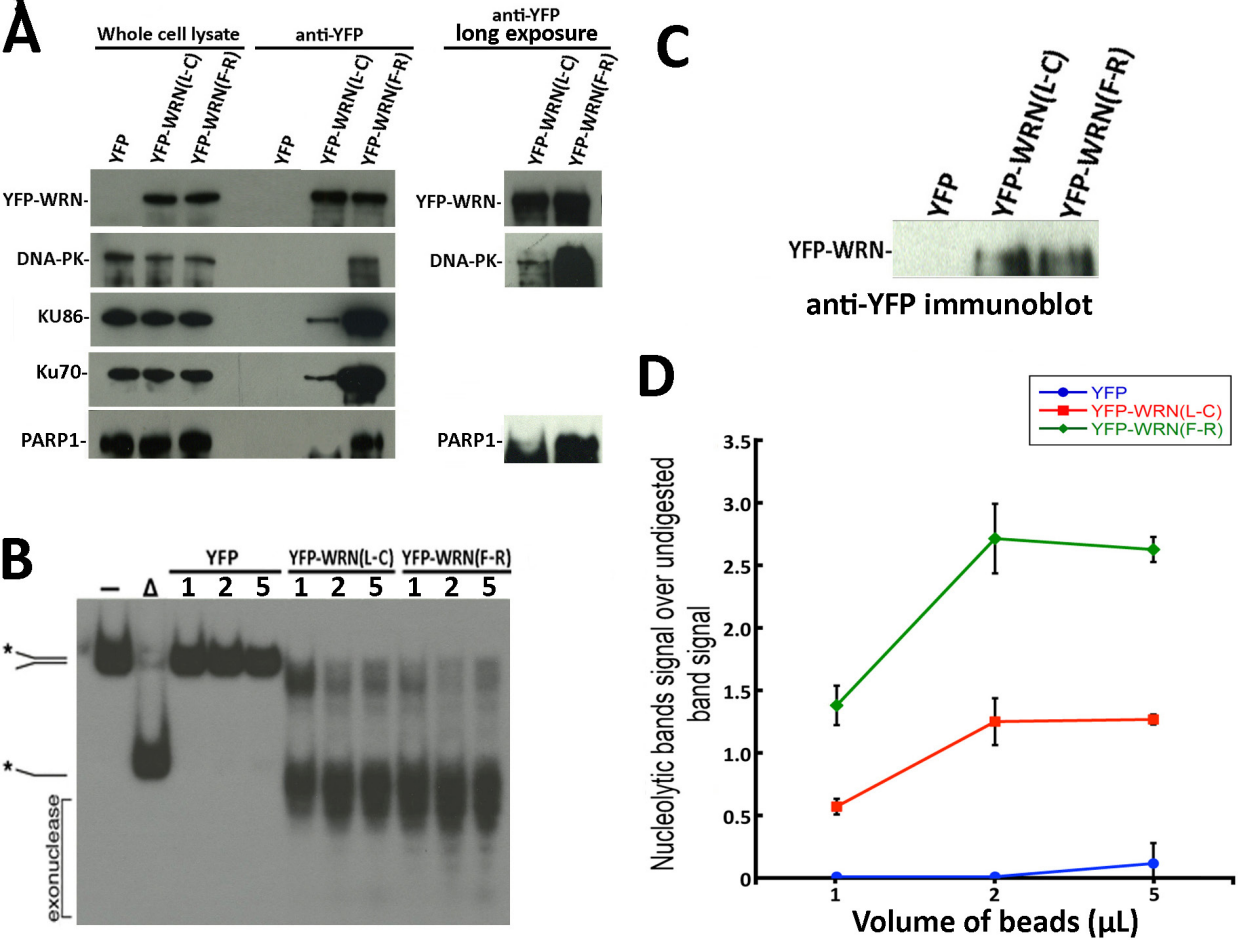
Immunoprecipitation of eYFP-WRN variants in WS fibroblasts (AG11395B) and evaluation of WRN exonluclease activity **A.** Representative Western blots against selected WRN-interacting proteins in WRN immunoprecipitation extracts from WS fibroblasts transfected with the eYFP-WRN(L-C) and eYFP-WRN(F-R) variants. Longer exposures of the immunoblots are shown on the right for DNA-PKc and PARP1. **B.** Evaluation of the exonuclease activity in eYFP (control), eYFP-WRN(L-C) and eYFP-WRN(F-R) immunoprecipitation extracts. The volumes of magnetic beads containing the eYFP-WRN variants and their interacting proteins were added as indicated for the enzymatic reactions. A radiolabeled splayed arms DNA was used as a WRN substrate to evaluate its exonuclease activity. The position of the different DNA processed forms and the nuclease fragments are indicated on the left. The autoradiogram shown represents 18 h of exposition. The asterisk indicates the radioactive strand. **C.** Western blot showing the levels of eYFP-WRN variants in transfected WS fibroblasts that were used for the enzymatic assays in B. **D.** Quantification of WRN exonuclease activity associated with eYFP-WRN(L-C) and eYFP-WRN(F-R) immunoprecipitation extracts. Activity ratios were calculated relative to the undigested band signal from the autoradiogram shown in B. The experiments were performed in duplicates. The error bars represent the SE.

We next analyzed the enzymatic activities of two different immunoprecipitated eYFP-WRN variants on a radioactive forked structure that showed the greatest difference in binding to DNA-PKc, KU86, KU70, and PARP1. The eYFP-WRN(F-R) and eYFP-WRN(L-C) variants were transfected in WRN-deficient WS fibroblasts (AG11395B). The eYFP expression vector was used as a control. The immunoprecipitations were performed as described in the preceding section except that Benzonase® and RNAse A were excluded from the lysis buffer to avoid contamination with these nucleases in the final step of our *in vitro* enzymatic assays. First, we reconfirmed the differential co-immunoprecipitation of DNA-PKc, KU86, KU70, and PARP1 on the eYFP-WRN(F-R) and eYFP-WRN(L-C) variants in WS fibroblasts (Figure 2A). As seen with the HEK293 cells, more DNA-PKc, KU86, KU70, and PARP1 were co-immunoprecipitated from the WS fibroblasts with the eYFP-WRN(F-R) variant than the eYFP-WRN(L-C) variant. Furthermore, we had to expose the immunoblots for a longer time to detect DNA-PKc and PARP1 in the eYFP-WRN(L-C) immunoprecipitates (Figure 2A). The amount of immunoprecipitated eYFP-WRN protein was evaluated by Western blotting for each variant to perform the *in vitro* enzymatic reactions with similar amounts of eYFP-WRN protein (Figure 2C). The enzymatic activities of the eYFP-WRN(F-R) variant were more efficient than the activity of the eYFP-WRN(L-C) variant (Figure 2B). The experiments were performed twice and the average enzymatic activity was approximately 1.5-fold higher with the eYFP-WRN(F-R) variant compared to the eYFP-WRN(L-C) (Figure 2D). Very little exonuclease and helicase activities were detected in the eYFP immunoprecipitate (Figure 2D). These results indicate that not only the immunoprecipitates of different eYFP-WRN variants tested contained different amount of DNA repair partner proteins but also exhibited different enzymatic activities *in vitro* toward a forked DNA structure.

### Cellular sensitivity to DNA damaging agents in the context of eYFP-WRN polymorphic variants

**Figure 3:**
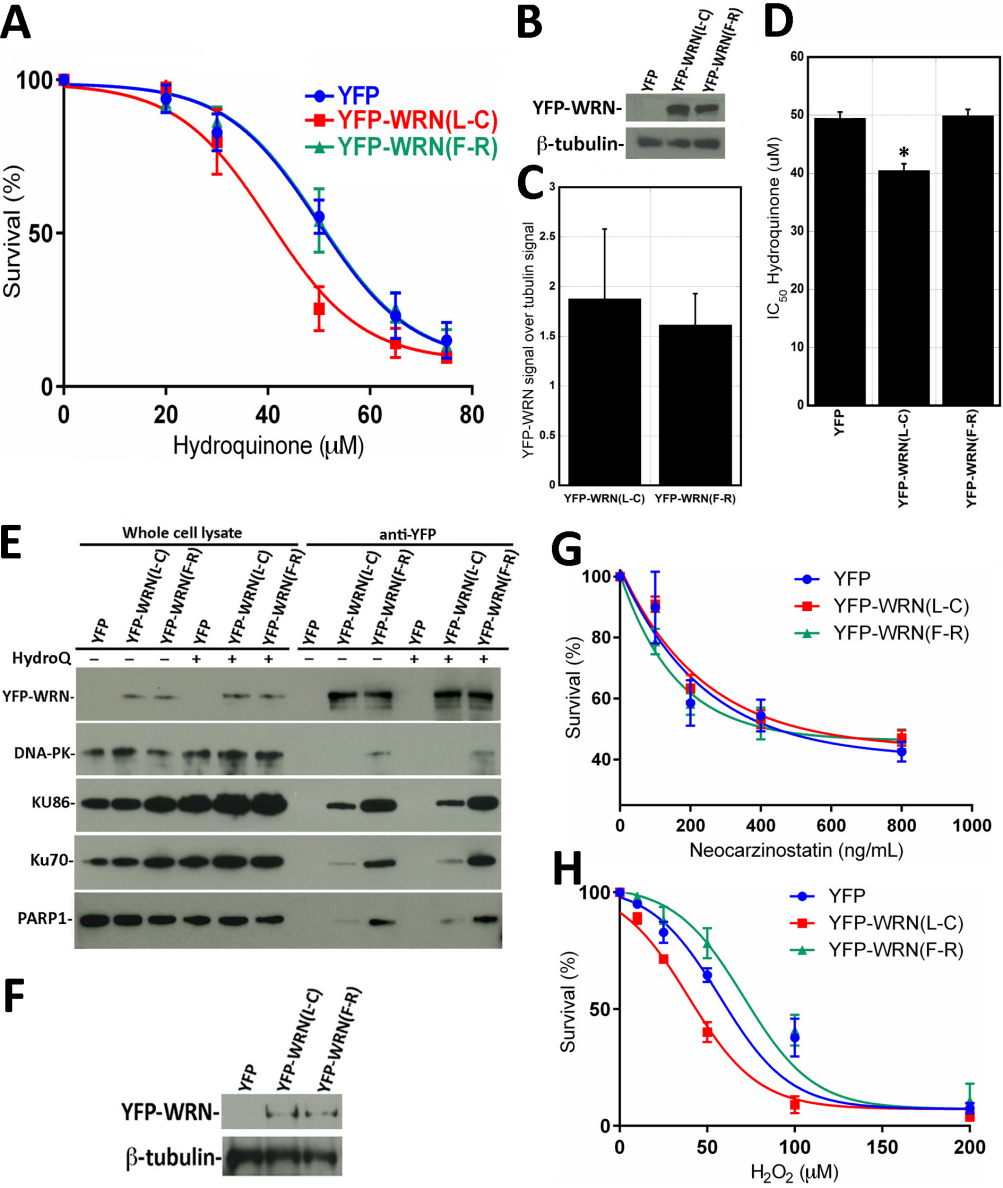
Dose-response curves of HEK293 cells expressing eYFP-WRN(L-C) and eYFP-WRN(F-R) variants exposed to genotoxic agents as determined by the sulforhodamine B colorimetric assay **A.** Graph showing the hydroquinone sensitivity of HEK293 cells expressing the eYFP-WRN variants as determined by the sulforhodamine B colorimetric assay. Experiments were repeated four times. Bars represent SE. **B.** Representative Western blot showing the expression of the eYFP-WRN variants in transfected HEK293 cells. β-tubulin was used as a loading control. **C.** Histogram presenting the ratio of eYFP-WRN signal over β-tubulin signal from Western blots. Bars represent SE. **D.** Histogram representing the IC50 of HEK293 cells expressing the eYFP-WRN(L-C) and (F-R) variants exposed to hydroquinone. Bars represent SEM. (*Unpaired student t-test P-value = 0.028 compared to the eYFP-WRN(F-R) variant). **E.** Representative Western blots against selected WRN-interacting proteins in WRN immunoprecipitation extracts in HEK293 cells treated with or without 40 μM hydroquinone (HydroQ). **F.** Example of a representative Western blot showing the expression of the eYFP-WRN variants in transfected HEK293 cells exposed to neocarzinostatin. ß-tubulin was used as a loading control. **G.** Cell survival curve of HEK293 cells exposed to neocarzinostatin. **H.** Cell survival curve of HEK293 cells exposed to hydrogen peroxide (H2O2). HEK293 cells were transfected with the indicated eYFP-WRN expressing vectors, exposed to the DNA damaging agents for 30 minutes with the indicated concentration range and allowed to recover for 48 hours in fresh media. The neocarzinostatin and hydrogen peroxide experiments were done in triplicates. Bars represent SE.

It has been reported that Chinese workers exposed to benzene exhibited different level of hematotoxicity depending on specific SNPs in the WRN gene [32]. We first determined the impact of the benzene metabolite hydroquinone on cell survival in HEK293 cells expressing either eYFP-WRN (L-C) or (F-R) variants. We determined by fluorescence microscopy that the transfection efficiency of HEK293 cells was > 80% with Effectene reagents (Qiagen). Transfected cells were treated overnight with different concentrations of hydroquinone, fixed, and stained with sulforhodamine B. As indicated by the survival curves in Figure 3A, cells transfected with the eYFP-WRN(L-C) variant were ∼19% more sensitive to hydroquinone than the eYFP-WRN(F-R) transfected cells. The protein transfection levels of eYFP-WRN variants were similar from one transfection experiment to another (Figure 3B and 3C). The IC_50_ for the eYFP-WRN(F-R) and eYFP-WRN(L-C) variants were 50.2 μM and 40.6 μM, respectively (Figure 3D; P-value = 0.011). Note that sequencing of the HEK293 exons encompassing the polymorphic amino acids under study revealed that such cells express an endogenous WRN with a Leu at position 1074 and a Cys at position 1367 (data not shown). Thus, overexpression of a polymorphic eYFP-WRN(L-C) protein in HEK293 cells had a negative effect on survival compared to cells overexpressing the eYFP-WRN(F-R) variant in an endogenous WRN(L-C) background.

To determine whether hydroquinone treatment could increase the interaction of the eYFP-WRN variants with DNA-PKc, KU86, KU70, and PARP1 proteins, immunoprecipitations were performed with extracts of hydroquinone-treated cells as well. As indicated in Figure 3E, more DNA-PKc, KU86, KU70, and PARP1 proteins were co-immunoprecipitated with the eYFP-WRN(F-R) variant than eYFP-WRN(L-C). (The supplementary Figure S1B presents a longer exposure to reveal the DNA-PKc protein in the eYFP-WRN(L-C) immunoprecipitate). The addition of hydroquinone, however, did not significantly change the amount of DNA-PKc, KU86, KU70, and PARP1 proteins co-immunoprecipitated with either eYFP-WRN variant (Figure 3E).

Hydroquinone is a genotoxic compound suspected to act as a cancer-causing agent. To determine whether double-strand breaks could affect the survival of transfected HEK293 cells, we treated these cells with the radiomimetic DNA double-strand cleaving agent neocarzinostatin. The amount of eYFP-WRN variants expressed in all HEK293 transfected cells was similar based on Western blot analyses (Figure 3F). As indicated in Figure 3G, no difference in resistance to neocarzinostatin could be detected between HEK293 cells transfected with the eYFP-WRN(L-C) or the eYFP-WRN(F-R) variant. Since hydroquinone can also induce an oxidative stress [36, 37], we examined the impact of hydrogen peroxide on transfected cells as well. As indicated in Figure 3H, cells transfected with the eYFP-WRN(L-C) variant were ∼43% more sensitive to hydrogen peroxide than the eYFP-WRN(F-R) transfected cells. The IC_50_ for the eYFP-WRN(F-R) and eYFP-WRN(L-C) variants were 74.4 μM and 42.3 μM, respectively. These results indicate that different polymorphic variants of the WRN protein influence how HEK293 cells respond to oxidative stress.

**Figure 4:**
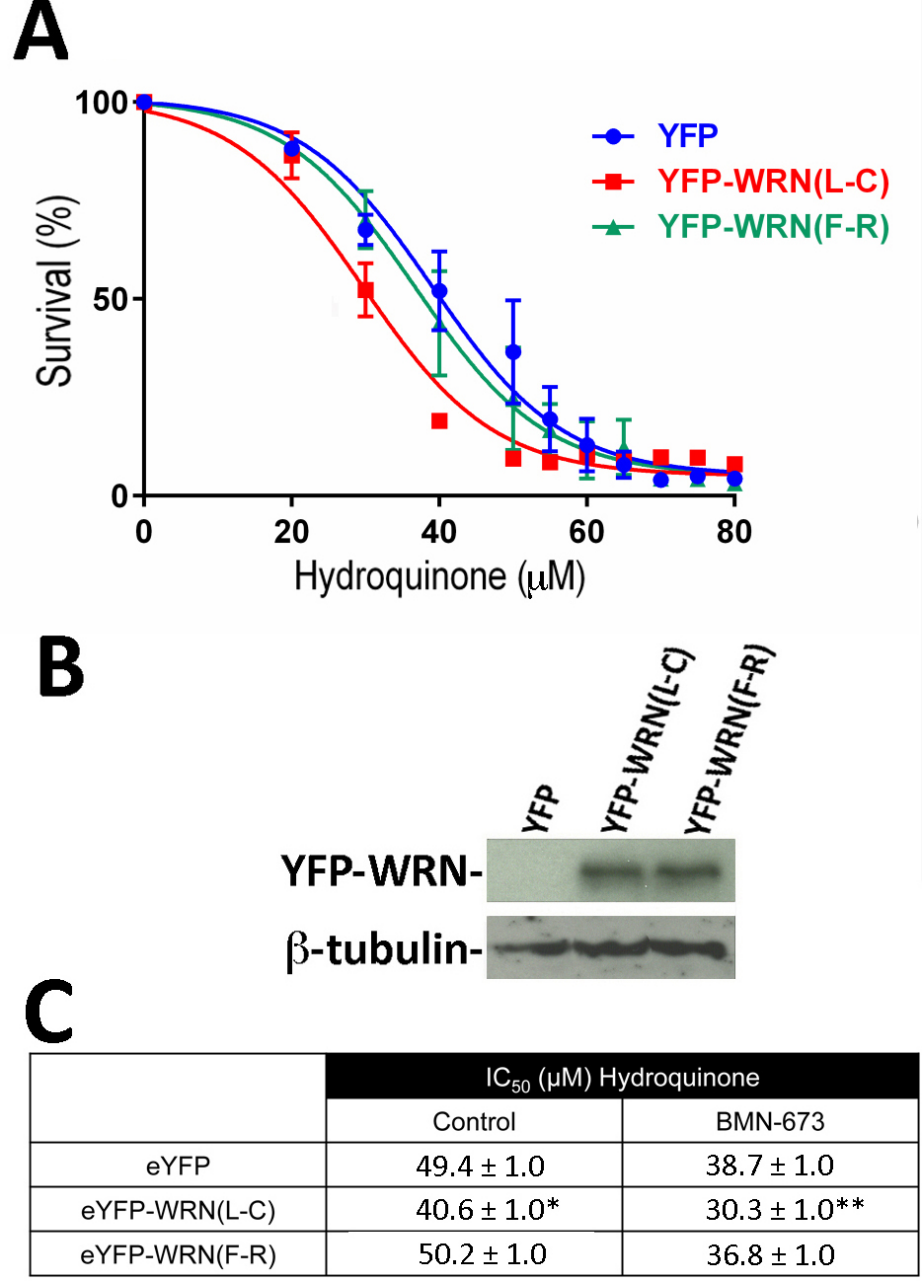
Dose-response curves of HEK293 cells expressing the eYFP-WRN(L-C) and eYFP-WRN(F-R) variants in presence of hydroquinone and PARP inhibition **A.** Cells were transfected with the indicated eYFP-WRN variants encoding vectors. Following overnight transfection, cells were treated one hour with 1 μM of the PARP1 inhibitor BMN-673 prior to exposure to increasing doses of hydroquinone for 24 hours. Hydroquinone dose-response curves were determined by the sulforhodamine B colorimetric assay. The experiments were performed in triplicates. Bars represent SE. **B.** Representative Western blot showing the expression of the eYFP-WRN variants in transfected cells. ß-tubulin was used as a loading control. **C.** Calculated IC50 (±SEM) of HEK293 cells expressing WRN variants from three different hydroquinone dose-response assays in the absence (control) or presence of the PARP1 inhibitor BMN-673. (*P-value = 0.011 and **P-value < 0.001 for eYFP-WRN(L-C) vs eYFP-WRN(F-R)).

Since a WRN/PARP1 complex is required for cellular survival upon oxidative stress [16], we examined the contribution of PARP-1 activation to the altered hydroquinone sensitivity observed between eYFP-WRN(L-C) and eYFP(F-R) expressing cells in additional survival assays. We thus treated the tranfected cells with the PARP1 inhibitor BMN-673 [38] for one hour prior to hydroquinone treatments (Figure 4A). The supplementary Figure S2 shows that the induction of poly(ADP)-ribosylation following hydroquinone-mediated DNA damage and PARP1 activation is totally inhibited by pretreatment of cells with BMN-673. As indicated in Figure 4, the PARP1 inhibitor sensitized all the transfected cells to the drug hydroquinone. The IC_50_ for the eYFP-WRN(F-R) and eYFP-WRN(L-C) variants were 36.8 μM and 30.3 μM, respectively. The amount of eYFP-WRN variants in all HEK293 transfected cells was similar based on Western blot analyses (Figure 4B). The difference between the hydroquinone's IC_50_ for the eYFP-WRN(F-R) and eYFP-WRN(L-C) variants was still ∼22% with the PARP1 inhibitor (Figure 4C).

**Figure 5:**
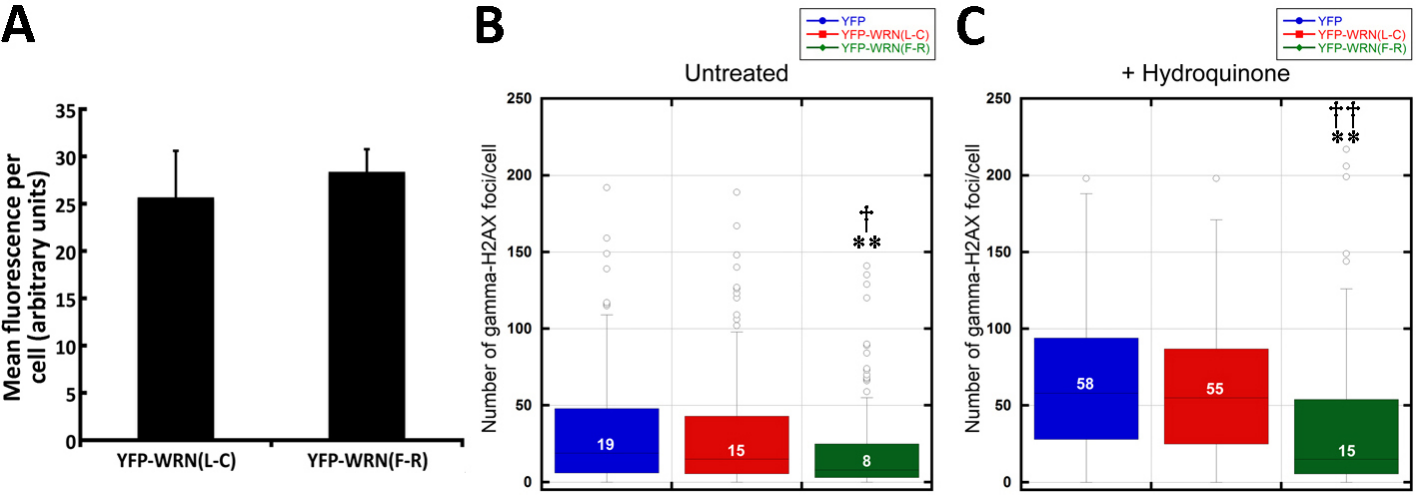
Box plots depicting the distribution of nuclear γ-H2AX foci in WS fibroblasts (AG11395B) expressing eYFP-WRN variants. **A.** Histogram representing the mean fluorescence intensity per tranfected WS fibroblast for each eYFP-WRN variant. FACS analyses were performed on triplicate transfections. **B.** WS fibroblasts were transfected with the indicated eYFP-WRN variants. Following overnight transfection, cells were fixed and processed for immunofluorescence analysis with an antibody against phosphorylated γ-H2AX. Tukey post ANOVA test P-values are shown (**P < 0.01 compared to eYFP transfected cells and †P < 0.05 compared to eYFP-WRN(L-C) transfected cells). **C.** Transfected WS fibroblasts were treated with 50 μM hydoquinone for four hours and then processed for immunofluorescence analysis with an antibody against phosphorylated γ-H2AX. Tukey post ANOVA test P-values are shown (**P < 0.01 compared to eYFP transfected cells and ††P < 0.01 compared to eYFP-WRN(L-C) transfected cells). The median number of γ-H2AX foci/cell is indicated in each box. All experiments were repeated three times.

We hypothesized that the overexpression of the eYFP-WRN(L-C) variant, which exhibits a lowest exonuclease activity than the eYFP-WRN(F-R) variant, may also result in the accumulation of unrepaired DNA damage intermediates in cells. To address this issue, we took advantage of the fact that eYFP-WRN expressing cells can be detected by fluorescence under an appropriate fluorescence microscope. More importantly, cells transfected with different eYFP-WRN variants can also be fixed for immunofluoresence analyses with an antibody against phosphorylated γ-H2AX. The number of γ-H2AX foci provides an estimate of DNA damage in eYFP-WRN fluorescing cells. To avoid the expression of the endogenous WRN protein in HEK293 that could impinge on the interpretation of the results, we transfected WS fibroblasts (AG11395B) with the two different eYFP-WRN variants. We first determined by FACS analysis that the average transfection efficiency for WS fibroblasts was ∼20% for all our constructs. The intensity of eYFP-WRN(L-C) and eYFP-WRN(F-R) fluorescence per WS fibroblast was also measured. As indicated in Figure 5A, the mean fluorescence per cell was not significantly different between eYFP-WRN(L-C) and eYFP-WRN(F-R) transfected WS fibroblasts. Figure 5B shows that the number of γ-H2AX foci per cell was significantly less in eYFP-WRN(F-R) expressing fibroblasts than in both eYFP and eYFP-WRN(L-C) expressing fibroblasts. The number of nuclear γ-H2AX foci significantly increased upon hydroquinone treatment (unpaired student t-test P < 0.01 for each transfected construct). However, eYFP-WRN(F-R) expressing cells showed a greater protection as the median number of nuclear γ-H2AX foci was significantly less than both eYFP and eYFP-WRN(L-C) expressing cells (Figure 5C). These results indicated that the eYFP-WRN(F-R) variant protected WS fibroblasts more efficiently than the eYFP-WRN(L-C) variant against hydroquinone-induced DNA damage.

**Figure 6:**
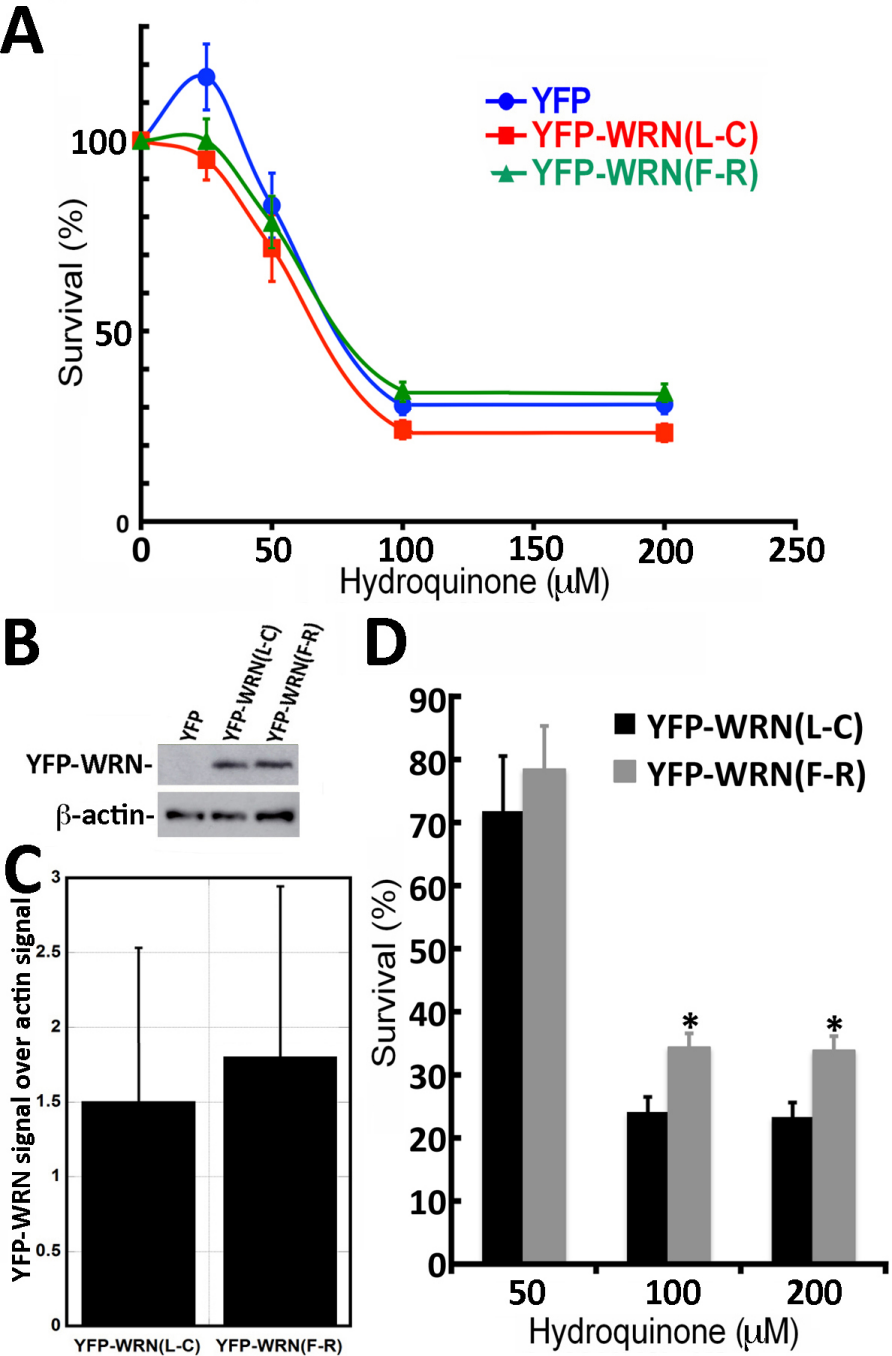
Dose-response curves of WS fibroblasts (AG11395B) expressing eYFP-WRN(L-C) and eYFP-WRN(F-R) variants exposed to hydroquinone as determined by the MTT assay **A.** Graph showing the hydroquinone sensitivity of WS fibroblasts expressing the eYFP-WRN variants as determined by MTT assay. Experiments were repeated 12 times. Bars represent SEM. **B.** Representative Western blot showing the expression of the eYFP-WRN variants in transfected WS fibroblasts. β-actin was used as a loading control. **C.** Histogram presenting the ratio of eYFP-WRN signal over β-actin signal from nine Western blots. Bars represent SE. **D.** Histogram representing the % survival of eYFP-WRN(L-C) and eYFP-WRN(F-R) transfected WS fibroblasts exposed to the indicated concentration of hydroquinone for 24 hours. Bars represent SEM for 12 transfections. (*Unpaired student t-test P-value < 0.01 compared to the eYFP-WRN(L-C) variant).

Even though the transfection efficiency in WS fibroblasts was much lower than in HEK293, we determined the impact of hydroquinone on cell survival in WS fibroblasts transfected with the eYFP-WRN(L-C) and eYFP-WRN(F-R) variants. Transfected fibroblasts were treated with different concentrations of hydroquinone overnight and cell survival was estimated by a MTT assay. As indicated by the survival curves in Figure 6A, there was a tendency for the eYFP-WRN(L-C) transfected fibroblasts to be more sensitive than the eYFP-WRN(F-R) transfected fibroblasts. The protein transfection levels of eYFP-WRN variants were similar from one transfection experiment to another (Figure 6B and 6C). The IC_50_ for the eYFP-WRN(F-R) and eYFP-WRN(L-C) variants were 75.5 μM and 67.7 μM, respectively (∼12% difference). Interestingly, there was a significant difference (P < 0.01) in cell survival between the eYFP-WRN(F-R) and eYFP-WRN(L-C) variants with the highest concentrations of hydroquinone treatments (Figure 6D). WS fibroblasts transfected with the eYFP-WRN(L-C) variant were more sensitive to 100 and 200 μM of hydroquinone than WS fibroblasts transfected with the eYFP-WRN(F-R) variant.

## DISCUSSION

Previous studies on purified WRN protein have indicated no significant difference with regards to exonuclease and DNA helicase activities when different polymorphic variants of the WRN protein were compared [34]. The two most common polymorphisms (most common in the general population) that were analyzed are single nucleotide polymorphisms (SNPs) affecting C-terminal amino acids 1074 (Leu to Phe) and 1367 (Cys to Arg) of the WRN coding sequence. Although purified WRN variants did not show enzymatic differences, we asked whether such variants interact with their cellular partners with the same affinity. By combining large-scale LC-MS/MS identification of affinity-purified eYFP-WRN-associated proteins and bioinformatics-based classification, this study represents the first large-scale proteomic identification of proteins binding onto different polymorphic variants of WRN and provides insights into the pathways that could be modulated by different coding SNPs in the WRN gene. We found that the WRN(L-C) polymorphism causes a significant drop in binding affinity to major DNA damage response factors such as DNA-PKc, KU70, KU86 and PARP1 when expressed in both HEK293 cells and WRN-deficient human fibroblasts derived from a WS subject.

Recently, it has been shown that WRN and KU80 interact directly together through a tandem of C-terminal KU-binding motifs (KBMs) [39]. The deletion of WRN C-terminal KBMs (located at residues 1399-1414 and 1415-1432) reduces the co-precipitation of KU80 by > 95%. This result underscores the importance of WRN C-terminal region for its association with KU proteins. Therefore, it is possible that WRN C-terminal polymorphisms could modify WRN affinity to KU proteins, which would explain the dramatic loss of KUs that we observed in eYFP-WRN(L-C) affinity-purification extracts. Several studies have indicated that the KU proteins increase the exonuclease activity of purified WRN protein [12, 13, 40]. DNA-PKc, in return, is known to phosphorylate the WRN protein [41] and to inhibit its exonuclease activity depending on the DNA substrates used in the enzymatic assays [42, 43]. However, the literature contains contradictory reports regarding whether the presence of DNA-PKc and KU proteins inhibit the exonuclease activity of the WRN protein [44] or release the inhibitory effect of DNA-PKc on WRN protein [42], especially in the context of PARP1 activation [45]. Using patient-derived WS fibroblasts, we found that the high affinity of the WRN(F-R) variant to DNA-PKc/KU complex correlates with high catalytic activity toward a forked DNA structure in contrast to the WRN(L-C) polymorphism. Because of the complex protein composition of the immunoprecipitates that were used in our enzymatic assays, it is impossible at this point to demonstrate unequivocally that interaction with the DNA-PKc/KU/PARP1 complex is actually up regulating WRN activities onto DNA and rule out the contribution of other nuclear proteins listed in the supplementary Table S1. This might be a challenging task since the dynamic function interplay of many DNA damage response factors is modified in the presence of poly(ADP-ribose), including WRN itself [46].

It has been hypothesized that subjects in the population who bear different coding SNPs in the WRN gene will behave differently in the presence of cytotoxic chemicals pollutants. For example, haplotypic analyses on different WRN coding SNPs indicated that Chinese workers exposed to benzene were more at risk of developing hematotoxicity depending on their polymorphic amino acids at both positions 1074 and 1367 [31, 32]. Indeed, it has been reported that WRN plays an important role in the protection of cells against the toxicity of benzene metabolites [47–49]. Thus, the difference between the polymorphic WRN variants in the population is likely due to how each variant interacts with their nuclear partners in cells and how efficient the different enzymatic activities of the WRN containing complexes are performing in cells. This is consistent with our observation that cells expressing the WRN(L-C) polymorphism are more sensitive to the benzene derived metabolite hydroquinone than WRN(F-R) variant in both HEK293 and WS cell lines. This also correlates with studies reporting that benzene-exposed workers with a WRN(L-C) haplotype are up to 20% more sensitive to benzene-hematotoxicity than workers with other WRN haplotypes [31].

Benzene metabolites, like reactive hydroquinones, are capable of binding to and damaging macromolecules including DNA, glutathione, tubulin, histones, topoisomerase II and other DNA-related proteins. Moreover, benzene metabolites may give rise to reactive oxygen species [36, 37] in addition to double-strand breaks [50]. We thus tested the impact of the radiomimetic drug neocarzinostatin and hydrogen peroxide on HEK293 cells transfected with the eYFP-WRN variants that co-immunoprecipitated the highest and the lowest amount of DNA-PKc, KU86, KU70, and PARP1. Importantly, the addition of the drug hydroquinone did not change the amount of DNA-PKc, KU86, KU70, and PARP1 co-immunoprecipitated with these variants even though the eYFP-WRN(L-C) variant was more sensitive to the drug than the eYFP-WRN(F-R) variant. The WRN variant containing a phenylalanine at position 1074 and an arginine at position 1367 co-immunoprecipitated more DNA-PKc, KU86, KU70, PARP1 in the presence or absence of hydroquinone than the WRN variant containing leucine at position 1074 and a cysteine at position 1367. These results indicate that WRN/DNA-PKc, WRN/KU or WRN/PARP1complexes pre-exist in untreated HEK293 cells. The amount of these cellular complexes is determined by the coding SNPs in cells. The survival tests with the radiomimetic drug neocarzinostatin and hydrogen peroxide indicated that eYFP-WRN(L-C) transfected HEK293 cells were more sensitive to hydrogen peroxide (i.e. oxidized DNA damage) than the eYFP-WRN(F-R) variant but no difference in cell survival between these variants were observed with neocarzinostatin (i.e. double-strand breaks). Importantly, deficient repair of oxidative DNA damage is commonly associated with premature aging and age-related diseases [51].

Unrepaired hydroquinone-induced DNA lesions lead to DNA breaks that can be monitored by the presence of phosphorylated γ-H2AX nuclear foci. Interestingly, eYFP-WRN(F-R) expressing WS fibroblasts exhibited significantly less γ-H2AX nuclear foci (i.e. double-strand breaks) than WS fibroblasts expressing the eYFP-WRN(L-C) variant. Since the eYFP-WRN(F-R) variant co-immunoprecipitated more DNA repair protein partners and exhibited higher nuclease activities than the eYFP-WRN(L-C) variant in WS fibroblasts, this suggests a better regulation and repair of DNA damage by eYFP-WRN(F-R)-containing protein complex in cells.

In summary, our results indicate that different polymorphisms in the WRN protein will impact hydroquinone genotoxicity through the modulation of DNA damage response-associated protein complex stoichiometry. Such results may imply that a WRN variant with a lower affinity for positive regulators during oxidative stress will have more deleterious effects on overall cell survival because of alterations in the fine-tuning of its enzymatic activity under DNA repair conditions. The higher number of γ-H2AX nuclear foci in WS cells expressing the eYFP-WRN(L-C) variant compared to the eYFP-WRN(F-R) variant is consistent with such findings. Finally, our results underscore the importance of functional haplotype-based analyses to appreciate the contribution of a group of proteins engaged together to produce particular phenotypic outcomes.

## MATERIALS AND METHODS

### Cell line and expression vectors

Human 293 embryonic kidney cells (HEK293; ATCC cat# CRL-1573) and the WS fibroblasts AG11395B (from the Coriell cell repositories) were maintained in Dulbecco's Modified Eagle Medium (DMEM) supplemented with 10% fetal bovine serum, penicillin (250 IU/mL), and streptomycin (250 μL/mL) at 37°C in atmosphere of 5% CO_2_. The eYFP control vector was purchased from Clontech (Mountain View, CA). The eYFP-WRN construct is described elsewhere [52]. The GenBank Accession code for the wild type cDNA sequence used in this study is NM_000553.4. The Leu and Cys at positions 1074 and 1367 of the WRN protein were respectively changed for Phe and Arg with the QuickChange site-directed mutagenesis kit from Stratagene (La Jolla, CA) to generate the different YFP-WRN polymorphic variants. These changes correspond to the single nucleotide polymorphisms rs1346044:T > C and rs1801195:G > T in the human WRN gene, respectively.

### eYFP-WRN Immunoprecipitation

HEK293 cells were seeded onto nine 150 mm cell-culture dishes and grown up to 80-90% confluency. Transfections were performed with the Effectene transfection reagent from Qiagen, Inc. (Mississauga, ON). The transfection efficiency in HEK293 cells was > 80%. All further steps were performed 24 h after the transfections on ice or at 4°C. Two PBS washes were carried out prior to the extraction (cell scraping) with 2 mL/plates of lysis buffer [40 mM HEPES pH 7.5, 120 mM NaCl, 0.3% CHAPS, 1 mM EDTA, Complete protease inhibitor cocktail (Roche Applied Science, Indianapolis, IN)]. Benzonase (25 U/mL) and RNase A (100 μg/mL) (EMD, Gibbstown, NJ) were also added in the buffer. Cells were kept on ice for 15 min and gently lysed for another 15 min on a rotating device. Cell extracts were centrifuged for 5 min at 3400 rpm to remove cellular debris. Immunoprecipitation experiments were performed using Dynabeads magnetic beads covalently coupled with Protein G (Invitrogen, Burlington, ON). The Dynabeads were washed once with 1 mL of 0.1 M sodium acetate buffer, pH 5.0, coated with 10-15 μg of mouse monoclonal anti-GFP antibody, which also recognizes eYFP protein (Roche Applied Science, Indianapolis, IN). The antibody-coupled Dynabeads were incubated for 1 h at room temperature with 1 mL of PBS containing 1% (w/v) BSA (Sigma-Aldrich, Oakville, ON) to block nonspecific antibody binding sites. The beads were finally washed three times with 2 mL of lysis buffer and added to the protein extract for 4 h incubation with gentle agitation in a cold room. (Protein extracts had been pre-cleared with empty Dynabeads for 45 min in a cold room before adding the anti-GFP coupled beads.) Samples were then washed three times with 2 vol of lysis buffer for 5 min. Protein complexes were eluted using 150 μL of 2X Laemmli sample buffer containing 5% β-mercaptoethanol and boiled for 5 min in a water bath. Proteins were resolved using 4-12% Criterion XT Bis-Tris gradient gel (Bio-Rad, Mississauga, Canada) and stained with Sypro Ruby (Bio-Rad, Mississauga, ON) according to the manufacturer's instructions. Images were acquired using the Geliance CCD-based imaging system (Perkin- Elmer, Shelton, CT).

### LC-MS/MS analysis

SDS-PAGE protein lanes corresponding to anti-eYFP immunoprecipitated extracts were cut into 33 gel slices per lane using a disposable lane picker (The Gel Company, San Francisco, CA). Gel slices were deposited into 96-well plates. In-gel protein digest was performed on a MassPrep liquid handling station (Waters, Mississauga, Canada) according to the manufacturer's specifications and using sequencing-grade modified trypsin (Promega, Madison, WI). Peptide extracts were dried out using a SpeedVac. Peptide extracts were separated by online reversed-phase (RP) nanoscale capillary LC (nanoLC) and analyzed by electrospray MS (ES MS/MS). The experiments were performed on a Thermo Surveyor MS pump connected to a LTQ linear ion trap mass spectrometer (Thermo Electron, San Jose, CA) equipped with a nanoelectrospray ion source (Thermo Electron, San Jose, CA). Peptide separation took place within a PicoFrit column BioBasic C18, 10 cm x 0.075 mm internal diameter (New Objective, Woburn, MA) with a linear gradient from 2% to 50% solvent B (acetonitrile, 0.1%formic acid) in 30 min, at 200 nL/min. Mass spectra were acquired using data-dependent acquisition mode (Xcalibure software, version 2.0). Each full-scan mass spectrum (400-2000 m/z) was followed by collision-induced dissociation of the seven most intense ions. The dynamic exclusion function was enabled (30 s exclusion), and the relative collisional fragmentation energy was set to 35%.

### Interpretation of tandem MS spectra

Tandem mass spectra were extracted by Mascot Deamon with Extract_MSN.exe. Charge state deconvolution and deisotoping were not performed. All MS/MS samples were analyzed using Mascot (Matrix Science, London, UK; version 2.4.1) and X! Tandem (The GPM, thegpm.org; version CYCLONE (2010.12.01.1)). Mascot was set up to search the Homo sapiens protein database (69137 entries) assuming the digestion enzyme trypsin. X! Tandem was set up to search a subset of database also assuming trypsin. Mascot and X! Tandem were searched with a fragment ion mass tolerance of 0.50 Da and a parent ion tolerance of 2.0 Da. Iodoacetamide derivative of cysteine was specified in Mascot and X! Tandem as a fixed modification. Oxidation of methionine was specified in Mascot as a variable modification. Pyro-glu from E of the n-terminus, s-carbamoylmethylcysteine cyclization (N-terminus) of the n-terminus and oxidation of methionine were specified in X! Tandem as variable modifications.

### Criteria for protein identification

Scaffold (version 4.2.0; Proteome Software, Inc., Portland, OR) was used to validate MS/MS-based peptide and protein identifications. Peptide identifications were accepted if they could be established at > 95.0% probability as specified by the Peptide Prophet algorithm [53]. Protein identifications were accepted if they could be established at > 95.0% probability and contained at least two identified peptides. Protein probabilities were assigned by the Protein Prophet algorithm [54]. Proteins that contained similar peptides and could not be differentiated based on MS/MS analysis alone were grouped to satisfy the principles of parsimony. For this study, a protein interacting with one of the WRN variants was considered positive if at least two MS/MS spectra could be assigned to a corresponding peptide. Proteins identified in a control HEK293 immunoprecipitation extract (expressing eYFP alone) were considered artifacts and removed from the final list of potential eYFP-WRN interacting proteins.

### Immunoblotting

The immunoprecipitated proteins were eluted from the DynaBeads, separated on SDS-PAGE, and then transferred onto 0.2 μm nitrocellulose membrane (Bio-Rad). After incubating 1 h with blocking solution (PBS-T containing 5% nonfat milk), the membrane was probed overnight at 4°C with either rabbit polyclonal antibodies against KU70, KU86, PARP1 (Santa Cruz Biotechnology, SantaCruz, CA), or a mouse monoclonal antibody against DNA-PKc (Oncogene Research Products, Cambridge, MA). The antibody against GFP (or eYFP) was purchased from BD Biosciences (Palo Alto, CA). After washing with PBS-T, species-specific horseradish peroxidase-conjugated secondary antibody was added for 2 h at room temperature. Signals were generated with Western Lightning Chemiluminescence reagent plus kit (GE Healthcare Limited, Piscataway, NJ).

### Exonuclease and helicase activities of immunoprecipitated eYFP-WRN

One 100 mm Petri dish of WS fibroblasts AG11395B (1.8×108 cells) was transfected with 6 μg of the eYFP or the eYFP-WRN plasmids with the TurboFectin 8.0 transfection reagent from Origene (Rockville, MD). The next day, eYFP and eYFP-WRN transfected cells were lysed in 1mL of a stringent buffer [50 mM Tris-HCl pH 8.0, 150 mM NaCl, 1.0% NP-40, 0.1% SDS, 0.5% Sodium deoxycholate, Complete protease inhibitor cocktail (Roche Applied Science, Indianapolis, IN)]. No benzonase and RNAse A was added in the lysis buffer. eYFP and eYFP-WRN were immunoprecipitated with 2 μg of an antibody against YFP and magnetic beads as described above. Immunoprecipitation was carried out for 2 h in a cold room. Beads containing the immune complexes were washed once with 1 mL of a buffer containing 20 mM Tris-HCl pH 8.0, 0.5 M NaCl, 1 mM EDTA, 0.5 mM DTT, 0,5% NP-40, 25% Glycerol, 0.2 mM PMSF. Beads were then washed twice with 2 mL of a buffer containing 20 mM Tris-HCl pH 8.0, 150 mM NaCl, 25% Glycerol, 0.5% NP-40, 0.05% Sodium deoxycholate, 0.005% SDS. Finally, beads were resuspended in 15 μL of buffer (25 mM Tris-HCl pH 8.0, 0.5 mM EDTA, 1 mM DTT, 0.05% NP-40, 25% glycerol). Resuspended beads containing the immune complexes (∼0.1 μg/μL of antibody) were diluted as indicated in the figures in assay reaction buffer (40 mM Tris-HCl pH 7.4, 4 mM MgCl_2_, 0.1 mg/mL BSA, 5 mM DTT, and 100 nM of splayed arms labeled on one DNA strand) [35]. The reaction was incubated for 20 min at 37°C, and stopped with one-fifth volume of Stop buffer (40% glycerol, 50 mM EDTA, 2% SDS, 3% xylene cyanol and 3% bromophenol blue). Reaction samples were loaded on a 6% PAGE/TBE 1X for autoradiography. The intensity of the bands representing the digested and undigested DNA structures on the autoradiogram was measured with Adobe Photoshop version 9.0.2.

### Drug treatments and sulforhodamine B colorimetric assay for HEK293 cells

HEK293 cells were transfected with the indicated constructs and allowed to grow for 24 hours. The next day, 10,000 cells were seeded per well on a 96-well plate and incubated at 37°C for 24 hours. Different concentrations of either the radiomimetic drug neocarzinostatin, the benzene metabolite hydroquinone, or hydrogen peroxide (Sigma-Aldrich, Oakville, ON) were added to the cells in triplicate and cells were then allowed to grow for an additional 18 hours. When indicated, transfected cells were treated for one hour with 1 μM of the PARP1 inhibitor BMN-673 (also known as talazoparib (Biomarin Pharmaceuticals), Selleckchem, Houston, TX) prior to the addition of different concentrations of hydroquinone. Cells were fixed with tricholoroacetic acid (10% w/v) and stained 30 min with sulforhodamine B as described [55]. The percentage of cell survival was calculated relative to untreated cells. The survival curves were plotted with GraphPad Prism 6.7 software via nonlinear regression. The Hill equation was used to estimate IC_50_ values, which are defined as the drug concentrations required to kill 50% of the cells.

### Immunofluorescence assays

WS fibroblasts (AG11395B) were transfected with 400 ng of eYFP, eYFP-WRN(L-C), or eYFP-WRN(F-R) constructs with Lipofectamine 2000 (Thermo Fisher Scientific Inc., Waltham, MA). The range of expression of the eYFP-WRN constructs in each transfected cell was evaluated by FACS analysis at the flow cytometry laboratory of the Centre de Recherche du CHU de Québec, (Québec City, Canada). For drug treatments, one day after the transfection, transfected cells were treated with 50 μM of hydroquinone for four hours. Cells were then washed twice in PBS, fixed with 2% paraformaldehyde in PBS for 10 min, washed with TBS and fixed with cold methanol (−20°C) for 5 min. Next, cells were washed once in TBS, permeabilized 5 min with PBS (0.2% Triton X-100) and washed three times 5 min with TBS. Then, cells were quenched with 0.1% sodium borohydride 5 min, washed once with TBS and blocked in PBS (10% goat serum and 1% BSA) one hour. Cells were then incubated one hour with an anti phospho-histone γ-H2AX ((Ser139) from EMD Millipore Corp., Billerica, MA, USA) diluted in 1% BSA/TBS. Cells were washed three times 5 min with TBS and incubated one hour with the appropriate secondary antibody (1% BSA/TBS) conjugated to a fluorophore (Alexa Fluor 568 (red) from Life Technologies). Cells were washed three times 10 min with TBS and coverslips were mounted onto slides with PBS-glycerol (90%) containing 1mg/ml paraphenylenediamine and 0.2 mg/ml of 4,6-diamidino-2-phenylindole (DAPI). For γ-H2AX scoring, images were obtained using a Leica CTR 6000 microscope. Number of foci per cell was automatically counted following background subtraction and deconvolution using Volocity software v 5.5 (Perkin-Elmer Improvision). Foci were scored according to intensity within a ≤ 0.7 μM radius. Approximately, 50 to 60 fluorescent cells/replicates were examined for each construct by two different persons. Transfection experiments were performed in triplicates and repeated thrice. Results are presented as box plots. One-way ANOVA followed by Tukey's HSD (honest significant difference) Test for post-ANOVA pair-wise comparisons were performed to determine significant differences with values of P < 0.05.

### Hydroquinone treatments and MTT assay for WS fibroblasts (AG11395B)

The reduction of tetrazolium salts is a reliable way to examine cell proliferation. The yellow tetrazolium MTT (3-(4, 5-dimethylthiazolyl-2)-2, 5-diphenyltetrazolium bromide) is reduced by metabolically active cells, in part by the action of dehydrogenase enzymes, to generate reducing equivalents such as NADH and NADPH. The resulting intracellular purple formazan can be solubilized and quantified by spectrophotometric means. Briefly, WS fibroblasts were transfected with the indicated constructs using the TurboFectin reagents (Origene) and allowed to grow for 24 hours. The next day, 4,000 cells were seeded per well on a 96-well plate and incubated at 37°C for six hours. Different concentrations of the benzene metabolite hydroquinone (Sigma-Aldrich, Oakville, ON) were then added to the cells in triplicate. Transfected cells were allowed to grow for an additional 24 hours. The next day, wells were rinsed with PBS and 200 μL of MTT reagent (0.25 μg/μL in fresh medium) was added to each well and plates were incubated at 37°C for four hours. The medium was removed and 150 μL of DMSO was added for spectrophotometric measurements. Absorbance was read at 540 nm and 670 nm.

## SUPPLEMENTARY MATERIALS FIGURES




