# Supplementary material for: Different non-synonymous polymorphisms modulate the interaction of the WRN protein to its protein partners and its enzymatic activities
Source: Oncotarget. 2016 Nov 14;7(52):85680–96. doi: 10.18632/oncotarget.13341 (PMC5349866; doi:10.18632/oncotarget.13341)
Supplement: Supplementary file 1 [file oncotarget-07-85680-s001.pdf]

Different non-synonymous polymorphisms modulate the interaction of the WRN protein to its protein partners and its enzymatic activities

Supplementary Material

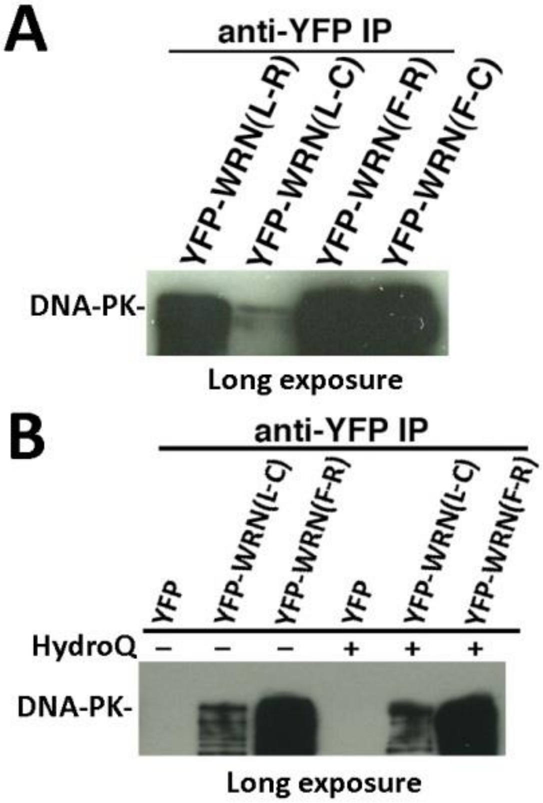

Figure S1: Validation of the presence of DNA-PKc in eYFP-WRN immunoprecipitation extracts by western blot analysis. An antibody directed against DNA-PKc was used to evaluate its abundance in HEK293-derived immunoprecipitation extracts relative to each eYFP-WRN variant. (**A**) DNA-PKc signal revealed in the immunoprecipitates of all eYFP-WRN variants by Western blotting after 45 min exposure. (**B**) DNA-PKc signal revealed in the immunoprecipitates of the eYFP-WRN(L-C) and eYFP-WRN(F-R) variants by Western blotting after 45 min exposure. Cells were treated with (+) or without (-) 40 µM hydroquinone (HydroQ) for 24 hours prior to the immunoprecipitation.

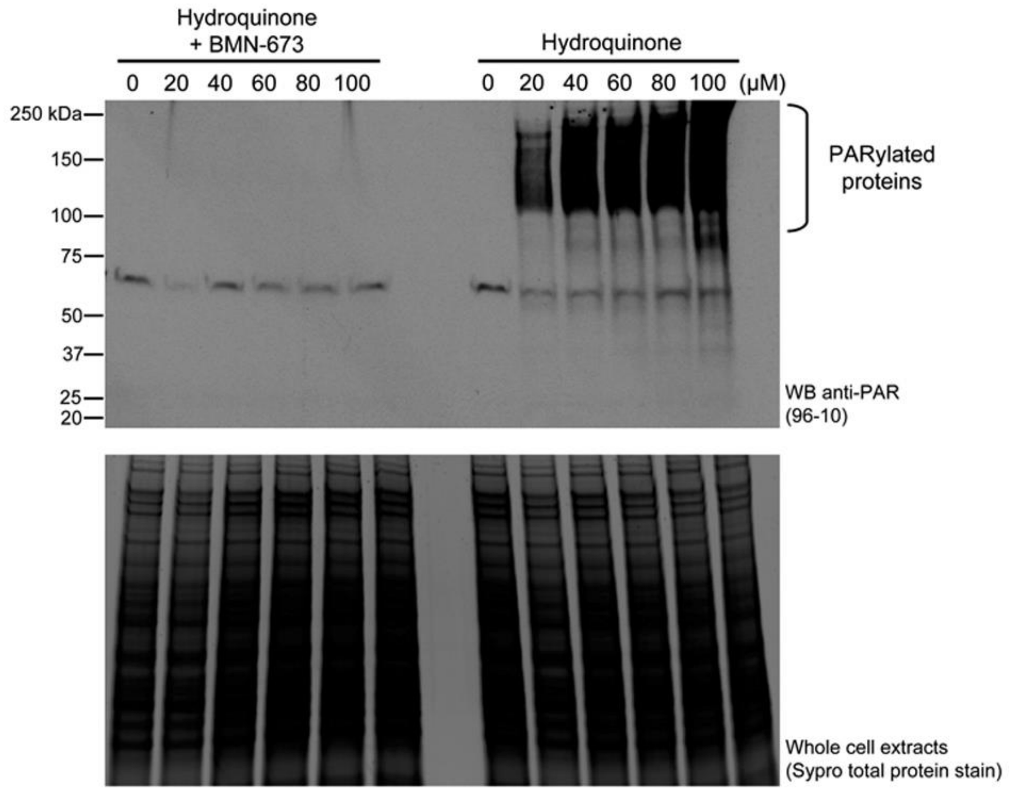

Figure S2: Western blot analysis of the protein PARylation profile in response to hydroquinone. HEK293 cells were incubated with or without the PARP1 inhibitor BMN-673 for 1 h prior to exposure to the indicated range of hydroquinone concentrations. The upper panel shows an immunoblot with a polyclonal antibody (clone 96-10) against PARylated proteins in HEK293 whole cell extracts. The lower panel shows a SYPRO protein staining of the cell extracts used for the Western blot shown above.
